# Relations between physical activity and hippocampal functional connectivity: Modulating role of mind wandering

**DOI:** 10.3389/fnhum.2022.950893

**Published:** 2022-10-03

**Authors:** Donglin Shi, Fengji Geng, Xiaoxin Hao, Kejie Huang, Yuzheng Hu

**Affiliations:** ^1^Department of Curriculum and Learning Sciences, Zhejiang University, Hangzhou, China; ^2^National Clinical Research Center for Child Health, Hangzhou, China; ^3^College of Information Science and Electronic Engineering, Zhejiang University, Hangzhou, China; ^4^Department of Psychology and Behavioral Sciences, Zhejiang University, Hangzhou, China

**Keywords:** physical activity, mind wandering, hippocampus, resting-state functional connectivity, cognitive function

## Abstract

Physical activity is critical for maintaining cognitive and brain health. Previous studies have indicated that the effect of physical activity on cognitive and brain function varies between individuals. The present study aimed to examine whether mind wandering modulated the relations between physical activity and resting-state hippocampal functional connectivity. A total of 99 healthy adults participated in neuroimaging data collection as well as reported their physical activity in the past week and their propensity to mind wandering during typical activities. The results indicated that mind wandering was negatively related to the resting-state functional connectivity between hippocampus and right inferior occipital gyrus. Additionally, for participants with higher level of mind wandering, physical activity was negatively related to hippocampal connectivity at left precuneus and right precentral gyrus. In contrast, such relations were positive at right medial frontal gyrus and bilateral precentral gyrus for participants with lower level of mind wandering. Altogether, these findings indicated that the relations between physical activity and hippocampal functional connectivity vary as a function of mind wandering level, suggesting that individual differences are important to consider when we aim to maintain or improve cognitive and brain health through increasing physical activity.

## Introduction

There is no doubt that physical activity is critical for maintaining cognitive and brain health throughout the lifespan (Hillman et al., 2008; Duzel et al., 2016). Physical activity is also known as a “medicine” due to its protective effects against cognitive declines (American College of Sports Medicine, 2009). Many studies have found that increased physical activity is associated with better cognitive performance, such as attention, visuospatial processing, cognitive control, and memory (Colcombe and Kramer, 2003; Donnelly et al., 2016; de Greeff et al., 2018). However, the neural mechanisms underlying the effect of physical activity on cognitive functions are still not clear. To address the issue, many neuroimaging studies have focused on the changes in neural structure and function associated with physical activity (Colcombe et al., 2004, 2006; Pontifex et al., 2011; Chen et al., 2019; Horowitz et al., 2020). Of all the documented brain structure and functional changes that responds to physical activity, robust changes have been observed in the hippocampus (Clark et al., 2012; Prakash et al., 2015; Rendeiro and Rhodes, 2018).

Hippocampus plays a critical role in integrating information during learning, memory consolidation, and retrieval (Tulving and Markowitsch, 1998; Lavenex and Lavenex, 2013). It has been shown in rodent models that increases in physical activity are associated with increased hippocampal neurogenesis (van Praag et al., 2005) and enhanced hippocampal long-term potentiation (van Praag et al., 1999; Liu et al., 2011). Moreover, physical activity has been found to modify the hippocampus at the molecular and morphological level (van Praag et al., 1999; Stranahan et al., 2007; Liu et al., 2011), and these modifications are thought to contribute to the improvements in hippocampal-dependent memory, learning, and other cognitive functions (Rendeiro and Rhodes, 2018).

Similarly, researchers also sought to understand the influence of physical activity on the hippocampus in humans (Erickson et al., 2009, 2011; Ruscheweyh et al., 2011; Chaddock et al., 2016). For instance, randomized controlled clinical trials indicated that people with higher physical activity or better fitness showed greater hippocampal volume (Chaddock et al., 2010, 2016). Such exercise-related changes in hippocampal volume were related to subsequent changes in memory performance (Erickson et al., 2009, 2011; Maass et al., 2015). The profound effect of physical activity on hippocampal structure has led researchers to explore how physical activity may impact hippocampal function, as measured by brain activation (Pereira et al., 2007; Burdette et al., 2010; Voelcker-Rehage et al., 2011), and functional connectivity between the hippocampus and other brain regions (Burdette et al., 2010; Chaddock et al., 2010; Voss et al., 2010; Prakash et al., 2011; Ikuta et al., 2019). For example, level of vigorous physical activity was significantly associated with right hippocampal-orbitofrontal connectivity during resting state (Ikuta et al., 2019). Furthermore, an intervention study indicated that 12-month physical exercise training in old adults increased the negative connection between prefrontal regions and anterior left hippocampus region during resting state (Voss et al., 2010). Another study found that the group who received 4-month aerobic exercise training showed greater connectivity between hippocampus and anterior cingulate cortex than the control group (Burdette et al., 2010).

However, whether individuals can get cognitive benefits from physical activity and how much benefit they can get vary from person to person (Madden et al., 1989; Rhodes and Smith, 2006; Fedewa and Ahn, 2011). The routes by which physical activity affects learning and memory are complex and may likely be moderated by many factors, including age, gender, health status, cognitive level, and numerous psychosocial factors (Tomporowski et al., 2011). For example, a meta-analysis study showed that children of different ages may get various degrees of cognitive benefits from physical activity, indicating that age may be an important moderator of the benefits of physical activity (Benjamin and Jennifer, 2003). Moreover, the relations of physical activity to cognitive functions and hippocampal volume differ by gender, with females obviously benefiting from physical activity to a greater extent than males (Barha et al., 2020). Therefore, before physical activity can be prescribed as “medicine” for the brain, it is important to better understand the factors contributing to this variation.

Mind wandering, as an important cognitive function supported by hippocampus (Faber and Mills, 2018; Mills et al., 2018), may moderate the relations of physical activity to learning and memory. Mind wandering is defined as thoughts that were not tied to concurrent perceptions, which occurs when the attention shifts away from the present situation to one’s inner thought (Singer, 1966; Smallwood et al., 2003). The resting-state functional connectivity within default mode network (DMN), within which the hippocampus is a node, has been shown to be closely related to self-generated thoughts that involved in mind wandering (Christoff et al., 2009; Andrews-Hanna, 2011; Xu et al., 2014; Ellamil et al., 2016; Mittner et al., 2016). For example, hippocampus was more activated during mind wandering than at rest, suggesting that mind wandering may induce the activation of hippocampus (Xu et al., 2014).

Furthermore, previous studies also suggested that mind wandering was related to future physical activity (Smallwood and Andrews-Hanna, 2013; Fanning et al., 2016). For instance, present-moment mind wandering was positively associated with future moderate-to-vigorous physical activity, indicating that the nature of one’s mind wandering may impact the ability to plan for or engage in the goal directed behavior (Fanning et al., 2016). Although few studies have directly tested the relations between physical activity and mind wandering, previous studies have repeatedly found that increasing physical activity improves sustained attention (Kumar et al., 2015; Luque-Casado et al., 2015; Ciria et al., 2017). For example, an ERP study found that higher aerobic fitness was related to neuroelectric activity, demonstrating a better overall sustained attention and the ability to allocate attentional resources (Luque-Casado et al., 2015). Therefore, mind wandering, as a cognitive activity supported by hippocampus, is also closely related to physical activity.

To sum, previous studies have shown that physical activity, mind wandering and hippocampal functions are related to each other (Voelcker-Rehage et al., 2011; Smallwood and Andrews-Hanna, 2013; Faber and Mills, 2018). Specifically, physical activity not only has positive impact on hippocampal functions, but also affects mind wandering (Luque-Casado et al., 2015; Chaddock et al., 2016). Additionally, mind wandering also affects hippocampal activity due to its role in inducing hippocampal activation (Xu et al., 2014; Ellamil et al., 2016). However, it is still unknown that whether mind wandering plays a role in the effect of physical activity on hippocampal functions. To provide insight into this question, the purpose of this study is to test whether mind wandering modulated the relations between physical activity and hippocampal functional connectivity measured during resting state. Specifically, we hypothesized that the relations between physical activity and hippocampal functional connectivity vary as a function of individual’s mind wandering level.

## Materials and methods

### Participants

A total of 105 college students were recruited from Zhejiang University at Hangzhou in China (mean age = 22.78, SD = 2.91, 49 female). Six participants were excluded from final analyses due to excessive head motion during fMRI scanning (*n* = 1) or incomplete questionnaires (*n* = 5). All participants were healthy without adverse health conditions, physical disabilities, or neurological disorders. This study was approved by the research ethics review board of Zhejiang University. Participants signed consent forms before participating in the study.

### Questionnaires

#### International physical activity questionnaire

Participants completed the short version of International Physical Activity Questionnaire (IPAQ) to measure their physical activity level during the last 7 days (Hagströmer et al., 2006). The items in the questionnaire were structured to measure the volume of vigorous-intensity activity, moderate-intensity activity, and walking per week. These activities were weighted by their energy requirements defined in MET (Metabolic Equivalent Task) to generate a score in MET-minutes, which is calculated by multiplying the MET score of an activity by the minutes performed. Total physical activity (MET-min/week) was calculated by the summation of vigorous, moderate activity, and walking in MET-minutes over a week. This summation score was used as a continuous variable to measure the physical activity level of participants in the current study.

#### Mind wandering questionnaires

The Mind Wandering Questionnaire (MWQ) (Mrazek et al., 2013) is a 5-item self-report scale, which was used to measure the propensity to mind wandering during typical activities (Cronbach’s α = 0.85). We used the Chinese version of the MWQ, which was verified to be a suitable tool to measure the trait level of mind-wandering (Luo et al., 2016). The questionnaire is a 6-point Likert scale, ranging from 1 (Never) to 6 (Always), with participants rating these items based on how often they experienced the particular situation (e.g., “I find myself listening with one ear, thinking about something else at the same time” or “I mind-wander during lectures or presentations”). Higher scores represent greater propensity to mind wandering.

### Imaging data acquisition and preprocessing

Participants were scanned in a Siemens 3.0T scanner (MAGNETOM Prisma, Siemens Healthcare, Erlangen, Germany) with a 20-channel coil in the Brain Imaging Science and Technology Center at Huajiachi Campus of Zhejiang University. They were asked to maintain their gaze at the fixation square in the center of the screen while they could blink as usual. Additionally, we explained to participants how subtle movements could affect data quality and asked them to remain as still as a statue to minimize head movement. The high-resolution structural images were acquired using a T1-weighted magnetization prepared-rapid gradient-echo sequence with the following parameters: TR = 2300 ms, TE = 2.32 ms, slice thickness = 0.9 mm, voxel size = 0.90 × 0.90 × 0.90 mm^3^, voxel matrix = 256 × 256, flip angle = 8°, and field of view = 240 mm^2^, duration of 7 min and 26 s. Then, a total of 480 whole-brain resting-state volumes were collected using a T2-weighted gradient echo planar imaging sequence: TR = 1000 ms, TE = 34 ms, slice thickness = 2.5 mm, voxel size = 2.50 × 2.50 × 2.50 mm^3^, voxel matrix = 92 × 92, flip angle = 50°, field of view = 230 mm^2^, slices = 52, and duration of 8 min.

The following steps were carried out to preprocess the data: (1) Slice timing correction and head motion correction were performed using AFNI.^1^ (2) Tissue segmentation was conducted to extract brains using SPM12.^2^ ANTs^3^ was used to co-register and normalize structural and functional images from original space to MNI space. (3) All functional images were spatially smoothed using a 5 mm full-width-at-half-maximum Gaussian kernel. (4) Nuisance variable regression was conducted using six-rigid head motion and their forward derivate as well as the first five principal components from white matter and cerebral spinal fluid (CSF) separately. (5) A band-pass filtering (0.01–0.1 Hz) was applied.

Since the resting-state functional connectivity could be influenced by small volume-to-volume head movements, we first calculated the framewise displacement (FD) of each volume to quantify the head motion. Any volume with FD ≥ 0.5 mm as well as 1 back and 1 forward volumes were scrubbed to minimize the effect of head motion. The mean FD of all participants included in the final statistical analyses was from 0.09 to 0.30 (mean FD = 0.153, SD = 0.038) with data length ≥ 7 min.

Then we performed resting-state functional connectivity analyses by using AFNI. We obtained the hippocampal seed regions from the Harvard–Oxford subcortical structure probabilistic atlas^4^ thresholded at 25%. With the uncal apex served as the border between anterior and posterior hippocampus (Duvernoy, 2005), the hippocampus was divided into anterior and posterior segments using manual identification of standard anatomical landmarks by using 1-mm MNI152 template.^5^ Therefore, left anterior, right anterior, left posterior, and right posterior hippocampus were used as seed regions. The functional connectivity between the time series of the seed regions and the other regions throughout the whole brain was calculated to generate the individual resting-state functional connectivity map (*r*-map). Then, by using Fisher’s r-to-z transformation, the *r*-maps were converted into z-maps to obtain the normally distributed values of the connectivity maps.

### Statistical analysis

Statistical analyses were conducted using 3dLME program within AFNI. To identify whether there were interactions between physical activity and mind wandering in predicting the functional connectivity between hippocampus and other brain regions, we added physical activity, mind wandering, and their interaction as independent variables in the fixed effect model. Since previous studies have found functional separation of hippocampus in different subregions and hemispheres (Strange et al., 1999; Iglói et al., 2010; Shipton et al., 2014; Robinson et al., 2015; Persson et al., 2018), we included the Subregions (anterior vs. posterior) and Hemispheres (left vs. right) as within-subject covariables. Random effects were also added to the model. The 3dClustSim in AFNI indicated that when *p*_uncorrected_ < 0.001, only clusters with a minimum of 23 voxel size were viewed as significant with multiple comparison correction (*p*_uncorrected_ < 0.001). We mainly reported the results involving physical activity, mind wandering, or both.

## Results

### Relation between physical activity and mind wandering

Table 1 showed the characteristics of participants who contributed both fMRI and questionnaires data. There was no significant correlation between physical activity and mind wandering (*p* = 0.094). Additionally, both of them were not significant related to mean FD (*r* = −0.048, *p* = 0.637; *r* = −0.004, *p* = 0.966). Therefore, mean FD was not included as covariate when we tested brain-behavioral relations below.

**TABLE 1:** Characteristics for participants who contributed both fMRI and questionnaires data.

Characteristics	Male			Female		
	Mean (SD)	Min	Max	Mean (SD)	Min	Max
Age (years)	22.71 (3.03)	18	30	22.75 (2.78)	18	29
Physical activity (MET-min/week)	2177.11 (1391.83)	66	7224	2544.78 (1475.21)	462	6813
Mind wandering	16.86 (4.92)	5	28	16.59 (5.08)	8	29

### Relation of physical activity and mind wandering to hippocampal functional connectivity

**FIGURE 1:**
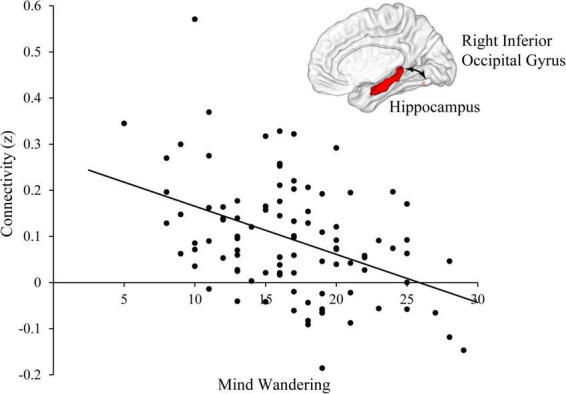
Mind wandering was negatively correlated with functional connectivity between hippocampus and right inferior occipital gyrus.

There was a significant main effect of mind wandering at right inferior occipital gyrus (Cluster size = 30; *x* = 45, *y* = −65, *z* = −14), suggesting that mind wandering was negatively related to the resting-state functional connectivity between hippocampus and right inferior occipital gyrus (Figure 1). Physical activity was not significantly related to hippocampal functional connectivity at any brain region. However, there were interactions between physical activity and mind wandering in predicting hippocampal functional connectivity at left precuneus, precentral gyrus, left superior frontal gyrus, and right medial frontal gyrus (Table 2).

**TABLE 2:** Interactions between physical activity and mind wandering.

Regions	Hemisphere	*F*	Cluster size	Peak MNI coordinates		
				x	y	z
Precuneus	left	21.925	113	−38	−65	36
Precentral gyrus	left	21.753	104	−35	12	41
Precentral gyrus	right	23.427	56	22	25	46
Superior frontal gyrus	bilateral	15.568	33	2	40	36
Medial frontal gyrus	right	17.104	33	−8	32	58

**FIGURE 2:**
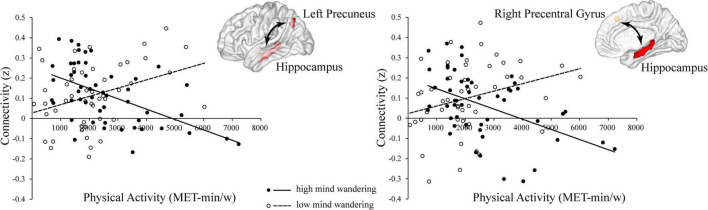
Negative relations between physical activity and hippocampal functional connectivity at left precuneus and right precentral gyrus in high mind wandering group.

**FIGURE 3:**
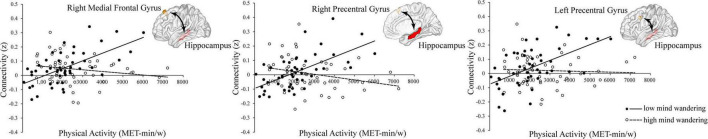
Positive relations between physical activity and hippocampal functional connectivity at right medial frontal gyrus, right precentral gyrus, and left precentral gyrus in low mind wandering group.

To understand these interactions, we separated all participants into high (48 subjects, 22 females, mean age = 22.71, SD = 2.75) and low groups (51 subjects, 27 females, mean age = 22.75, SD = 3.05) according to the mean scores of their mind wandering (i.e., 16.73). Then, for each group, we conducted whole-brain search analyses to test whether physical activity was related to hippocampal functional connectivity at each brain region showing significant interaction. The results indicated that in high mind wandering group, physical activity was negatively related to hippocampal functional connectivity at left precuneus and right precentral gyrus (Figure 2). In contrast, in low mind wandering group, there were positive correlations between physical activity and hippocampal functional connectivity at right medial frontal gyrus, right precentral gyrus, and left precentral gyrus (Figure 3 and Table 3). However, the relations between physical activity and hippocampal functional connectivity at left superior frontal gyrus did not survive from multiple comparison correction in both low and high mind wandering groups.

**TABLE 3:** Mind wandering modulated the relationship between physical activity and hippocampal functional connectivity.

	*Z*	Cluster size	Peak MNI coordinates			Polarity
			x	y	z	
**Regions showing significant connectivity in high mind wandering group (*N* = 48)**						
Left precuneus	−3.974	38	−38	−63	38	-
Right precentral gyrus	−4.026	26	25	30	46	-
**Regions showing significant connectivity in low mind wandering group (*N* = 51)**						
Right medial frontal gyrus	4.553	69	−8	35	56	+
Right precentral gyrus	3.988	52	22	20	61	+
Left precentral gyrus	4.489	33	−35	12	38	+

## Discussion

The current study aimed to examine whether mind wandering modulated the relations between physical activity and hippocampal functional connectivity. First, we found that mind wandering was negatively related to the resting-state functional connectivity between hippocampus and right inferior occipital gyrus. Additionally, we found there was significant interaction between physical activity and mind wandering in predicting the resting-state hippocampal functional connectivity at left precuneus, precentral gyrus, left superior frontal gyrus, and right medial frontal gyrus. Specifically, for participants with higher level of mind wandering, there was negative relation between physical activity and hippocampal functional connectivity at left precuneus and right precentral gyrus. For participants with lower level of mind wandering, physical activity was positively related to hippocampal functional connectivity at right medial frontal gyrus and bilateral precentral gyrus. These findings supported our hypothesis that mind wandering modulated the relations between physical activity and hippocampal functional connectivity.

### Interactions between physical activity and mind wandering in hippocampal functional connectivity

Our study found the significant interaction between physical activity and mind wandering in predicting hippocampal functional connectivity at left precuneus, right medial frontal gyrus, and bilateral precentral gyrus. These brain regions, similar to hippocampus, are closely related to physical activity or/and mind wandering (Schneider et al., 2009; Vago and David, 2012; Boccia et al., 2015; Christoff et al., 2016; Thielen et al., 2016). For example, the DMN, which includes precuneus and medial frontal gyrus as nodes, has been shown to be positively activated during mind wandering (Buckner et al., 2008; Andrews-Hanna, 2011). Additionally, a meta-analysis study showed that compared with controls, the meditators, who would experience more mind wandering, had greater activation in precuneus, precentral gyrus, and medial frontal gyrus during meditation (Boccia et al., 2015).

Meanwhile, numerous studies also found that during both resting and task states, people with higher physical activity or fitness showed greater activation at precuneus, precentral gyrus, and medial frontal gyrus than people with lower physical activity or fitness (Casey et al., 2000; Schneider et al., 2009; Voelcker-Rehage et al., 2010; Smith et al., 2011; Kimura et al., 2013; Thielen et al., 2016). For example, compared to the controls, people who received aerobic physical activity intervention showed greater activation at precuneus (Thielen et al., 2016), precentral gyrus (Voelcker-Rehage et al., 2010; Smith et al., 2011), and medial frontal gyrus (Casey et al., 2000; Kimura et al., 2013) during performing tasks that were highly demanding. Altogether, previous studies suggest that left precuneus, right medial frontal gyrus, and bilateral precentral gyrus may co-activate with hippocampus to support mind wandering and the functions of these brain regions may also be influenced by physical activity.

Furthermore, we found that the relations between physical activity and hippocampal functional connectivity vary as a function of mind wandering level. Previous studies have indicated that maintaining appropriate mind wandering level is critical in our daily life (Smallwood and Andrews-Hanna, 2013). Too much mind wandering may cause us to be dissociated from the external environment, which may result in deteriorated task performance, such as the performance in working memory task (McVay and Kane, 2010) and reading comprehension task (Smallwood et al., 2008; Unsworth and McMillan, 2013). However, a certain amount of mind wandering is also beneficial in some situations. For example, we also benefit from mind wandering in tasks or environments that require divergent thinking, such as creativity (Baird et al., 2012), problem solving (Smeekens and Kane, 2016), and successful management of long-term goals (Smallwood et al., 2013). Therefore, mind wandering enables us to reach balance in allocating attention to processing internal and external stimuli.

Previous studies also indicated that physical activity is related to the allocation of attentional resources (Hillman et al., 2006; Luque-Casado et al., 2015; Stillman et al., 2018). For example, physical activity had a larger effect on behavioral performance during task switch vs. repeat (Hillman et al., 2006), suggesting that physical activity affects the processing of information in tasks that have great demands on attentional resource (Kramer et al., 1999; Colcombe and Kramer, 2003). Additionally, another ERP study indicated that fitness was positively related to the neuroelectric activity that measures sustained attention, suggesting that individuals with higher fitness are more capable of allocating attentional resources over time (Luque-Casado et al., 2015).

Therefore, in terms of the findings that individuals with high vs. low mind wandering showed opposite relations between physical activity and hippocampal functional connectivity, we propose that physical activity may help individuals maintain appropriate level of mind wandering through affecting the interactions between hippocampus and other brain regions. Specifically, for people with lower level of mind wandering, physical activity may help intensify the functional connectivity between brain regions within networks of mind wandering. In contrast, for people with higher level of mind wandering, physical activity may reduce the connectivity between regions of brain networks that support mind wandering (Stillman et al., 2018). Although such interpretation needs more studies to verify, we further propose that when physical activity is considered as a kind of “medicine,” individual differences are important to consider. In other words, the effect of physical activity may vary substantially between different people.

### Main effect of mind wandering at inferior occipital gyrus

We found that mind wandering level was associated with the functional connectivity between hippocampus and right inferior occipital gyrus. The occipital gyrus participates in processing visual information and has been suggested to be important for object recognition (Gauthier et al., 2000; Sato et al., 2014; Jacques et al., 2019). Visual information is preliminarily processed and integrated in inferior frontal gyrus and then reprocessed in higher-level cognitive systems, such as the memory center for which hippocampus is a critical structure (Stone, 1983; Eichenbaum et al., 1992; Andersen et al., 2006). It has been suggested that compared to people with lower level of mind wandering, people with higher level of mind wandering focus more on processing internal information, but ignore external information or process it at superficial level without sending them to higher-order cognitive systems (i.e., hippocampal memory system) (Hasenkamp et al., 2012). As a result, for people with higher level of mind wandering, the connectivity between hippocampus and inferior occipital gyrus may become weaker compared to the ones with lower level of mind wandering.

### Strengths and limitations

Previous studies showed that physical activity, hippocampal function, and mind wandering were related to each other. Our study contributed to establishing the modulating role of mind wandering in the relations between physical activity and resting-state hippocampal functional connectivity. However, the current study still had several limitations. First, the study design did not allow us to test the causal relations between physical activity, mind wandering, and hippocampal functions. Additionally, physical activity and mind wandering were measured by self-reported, which is subjective and maybe biased (Randy et al., 2003). Therefore, future studies need to include more objective measurements and use study designs that allow us to test the causal effect of physical activity on mind wandering and the related brain functions.

## Conclusion

To summarize, this study established the modulating role of mind wandering on the relations between physical activity and resting-state hippocampal functional connectivity at precuneus, precentral gyrus, and medial frontal gyrus. Specifically, for individuals with higher level of mind wandering, physical activity was negatively related to hippocampal functional connectivity at left precuneus and right precentral gyrus; in contrast, for individuals with lower level of mind wandering, physical activity was positively related to hippocampal functional connectivity at right medial frontal gyrus and bilateral precentral gyrus. We interpreted such findings as that physical activity may help maintain an appropriate level of mind wandering by affecting the interaction between hippocampus and other brain regions. Therefore, the current study provides insight into the variations between individuals on the relations between physical activity and brain functions, implying that individual differences are important to consider when we aim to maintain or improve neurocognitive health through increasing physical activity.

## Data availability statement

The raw data supporting the conclusions of this article will be made available by the authors, without undue reservation.

## Ethics statement

The studies involving human participants were reviewed and approved by Zhejiang University. The patients/participants provided their written informed consent to participate in this study.

## Author contributions

DS drafted the initial manuscript, carried out data analysis, interpreted results, and critically revised the manuscript for important intellectual content. FG conceived and designed the study, contributed to acquisition, analysis and interpretation of the data, drafted, reviewed, and critically revised the manuscript for important intellectual content. XH contributed to data collection and critically revised the manuscript for important intellectual content. KH and YH contributed to conception of the study and critically revised the manuscript for important intellectual content. All authors approved the final manuscript as submitted.

## Footnotes

1
https://afni.nimh.nih.gov/

2
https://www.fil.ion.ucl.ac.uk/spm/

3
http://stnava.github.io/ANTs/

4
https://fsl.fmrib.ox.ac.uk/fsl/fslwiki/Atlases

5
http://www.bic.mni.mcgill.ca/ServicesAtlases/ICBM152NLin2009
